# Associations of polygenic risk scores differentiating attention-deficit hyperactivity disorder from autism spectrum disorder with cognitive and cortical alterations in Schizophrenia patients

**DOI:** 10.1007/s00787-024-02549-w

**Published:** 2024-08-07

**Authors:** Ayumi Kuramitsu, Kazutaka Ohi, Toshiki Shioiri

**Affiliations:** 1 Department of Psychiatry, Gifu University Graduate School of Medicine, 1-1 Yanagido, Gifu, Gifu, 501- 1194 Japan; 2 Department of Internal Medicine, Kanazawa Medical University, Ishikawa, Japan

**Keywords:** Schizophrenia, Autism spectrum disorder, Attention-deficit hyperactivity disorder, Polygenic risk score, Cortical structure

## Abstract

Schizophrenia (SCZ) is a clinically and genetically heterogeneous disorder that shares genetic factors with autism spectrum disorder (ASD) and attention-deficit hyperactivity disorder (ADHD). A genome-wide association study (GWAS) differentiating ADHD from ASD was performed recently. In this study, we investigated whether polygenic risk scores (PRSs) differentiating ASD from ADHD are associated with cognitive impairments and alterations in cortical structures in SCZ patients. Based on the GWAS data (9,315 ASD and 11,964 ADHD patients), PRSs differentiating ADHD from ASD (indicating a greater risk of ADHD and a lower risk of ASD) were calculated for SCZ patients (*n* = 168). Cognitive performance, including verbal comprehension (VC), perceptual organization (PO), working memory (WM), and processing speed (PS), was assessed using the WAIS-III (*n* = 145). The surface areas and cortical thicknesses of 34 bilateral brain regions were extracted using FreeSurfer (*n* = 126). We examined the associations of these PRSs with cognitive performance and cortical structures in SCZ patients. Among the four cognitive domains, a higher PRS, indicating a greater risk of ADHD, was associated with impaired WM in SCZ patients (*beta*=-0.21, *p* = 0.012). A lower PRS, indicating a greater risk of ASD, was associated with decreased surface areas of the left medial orbitofrontal (*beta* = 0.21, *p* = 8.29 × 10^− 4^), left entorhinal (*beta* = 0.21, *p* = 0.025), left postcentral (*beta* = 0.18, *p* = 7.52 × 10^− 3^), right fusiform (*beta* = 0.17, *p* = 6.64 × 10^− 3^), and left fusiform cortices (*beta* = 0.17, *p* = 7.77 × 10^− 3^) in SCZ patients. A higher PRS, indicating a greater risk of ADHD, was associated with decreased cortical thickness in the bilateral transverse temporal regions (left, *beta*=-0.17, *p* = 0.039; right, *beta*=-0.17, *p* = 0.045). Our study revealed a relationship between genetic factors that differentiate ADHD patients from ASD patients and both cortical structure and cognitive performance in SCZ patients. These findings suggest that the heterogeneity of SCZ might be partly derived from genetic factors related to neurodevelopmental and psychiatric disorders other than SCZ.

## Introduction

Schizophrenia (SCZ) is a common, clinically and genetically heterogeneous psychiatric disorder. The lifetime morbidity rate of SCZ patients ranges from 0.5 to 1.0%, and SCZ is characterized by cognitive impairments; positive symptoms, such as hallucinations and delusions; and negative symptoms, such as blunted affect, poor rapport, and stereotyped thinking. Autism spectrum disorder (ASD) and attention-deficit hyperactivity disorder (ADHD) are common neurodevelopmental disorders. ASD affects approximately 1.0% of individuals [1] and is characterized by impaired social interactions and communication, along with restricted and repetitive behavior from early development. ADHD affects approximately 5.0% of children and 2.5% of adults and is characterized by greater inattention and impulsivity compared to individuals with typical development. Although these disorders are distinguished as different psychiatric and neurodevelopmental disorders based on their clinical symptoms according to international diagnostic criteria, *the Diagnostic and Statistical Manual of Mental Disorders* (DSM) and *the International Classification of Diseases* (ICD), the prevalence of ASD in individuals with SCZ has been reported to range from 3.4 to 52.0% [2], and the prevalence of childhood and adult ADHD in individuals with SCZ ranges from 10.0 to 57.0% [3]. This suggests considerable clinical heterogeneity among individuals with SCZ.

SCZ [4–7], ASD [8], and ADHD [9] are highly heritable disorders, with estimated heritability rates of approximately 60–90%. Large-scale genome-wide association studies (GWASs) conducted by the SCZ, ASD, and ADHD Working Group of the Psychiatric Genomics Consortium (PGC) and the Lundbeck Foundation Initiative for Integrative Psychiatric Research (iPSYCH) have identified 287, 5, and 12 distinct genome-wide-significant loci for SCZ [10], ASD [11] and ADHD [12], respectively. These disorders share a partially overlapping genetic etiology [11–15]. Moderately positive genetic correlations have been found between SCZ and ASD (*r*_G_=0.21) [11] and ADHD (*r*_G_=0.12) [12].

Moreover, a recent GWAS from the iPSYCH and the PGC differentiating the two neurodevelopmental disorders (ASD and ADHD) in 9,315 individuals with ASD and 11,964 individuals with ADHD identified five neurodevelopmental disorder-specific loci [16]. The genetic factor differentiating ADHD from ASD (indicating a higher risk of ADHD and a lower risk of ASD) was positively correlated with ADHD (*r*_G_=0.59) and negatively correlated with ASD (*r*_G_=-0.60), with no significant correlation with SCZ (*r*_G_=-0.03) [16]. SCZ and ADHD have shown negative genetic correlations with cognitive function [SCZ; *r*_G_=-0.46 [17], ADHD; *r*_G_=-0.41 [12]], whereas ASD has demonstrated positive genetic correlations with cognitive function (*r*_G_=0.20) [11]. Furthermore, the genetic factor differentiating ADHD from ASD was negatively correlated with several cognitive traits, including years of schooling (*r*_G_=-0.67) and intelligence (*r*_G_=-0.59) [16].

Cortical surface area and thickness are associated with increased brain function and serve as independent morphological markers of cortical structure. It has been suggested that different developmental mechanisms underlie surface area expansion and increases in thickness [18]. The SCZ, ASD, and ADHD Working Groups of the worldwide ENIGMA (Enhancing Neuro Imaging Genetics through Meta-Analysis) consortium have investigated disorder-specific abnormalities in regional surface area and cortical thickness [19–21]. Compared with healthy participants, individuals with SCZ exhibit a widespread smaller cortical surface area and thinner cortex [19]. Increased cortical thickness in the frontal cortex and decreased cortical thickness in the temporal cortex have been observed in individuals with ASD, with no differences in surface area compared to healthy participants [21]. Compared with healthy individuals, individuals with ADHD have a widespread decrease in cortical surface area and a partially thinner cortex, including the fusiform gyrus, precentral gyrus, and temporal pole [20]. Moreover, adults with ADHD exhibit lower cortical thickness in the orbitofrontal, inferior frontal and cingulate areas than adults with ASD [22]. Large-scale GWASs for regional cortical surface area and thickness have identified 369 genome-wide nominally significant loci related to cortical structures [18]. 26% of the variation in average cortical thickness and 34% of the variation in total surface area can be explained by common single-nucleotide polymorphisms (SNPs) [18]. Although genetic correlations of regional cortical structures with the risk of SCZ, ASD, and ADHD have been identified, e.g., SCZ and the medial orbitofrontal area (*r*_G_=0.10), ADHD and the fusiform area (*r*_G_=-0.14), and ASD and the fusiform area (*r*_G_=-0.15) [18], no study has yet investigated the genetic correlations between the genetic factor differentiating ADHD from ASD and brain cortical structures or whether this genetic factor influences cognitive functions and brain cortical structures in patients with SCZ.

Given the clinical and genetic heterogeneity of SCZ and its significant overlap with ASD and ADHD, our study focuses specifically on SCZ to better understand the distinct genetic contributions to cognitive impairments and cortical abnormalities within this population. By isolating SCZ as the primary condition, we aim to elucidate the unique and shared genetic underpinnings that differentiate it from other neurodevelopmental disorders.

The significance of this study lies in its potential to unravel the specific genetic influences on cognitive impairments and cortical abnormalities in SCZ. By exploring the polygenic risk scores (PRSs) differentiating ADHD from ASD, we aim to contribute to the understanding of the genetic architecture that impacts cognitive functions and brain structures in SCZ. This could lead to more precise diagnostic tools and personalized treatment strategies, ultimately improving outcomes for individuals with SCZ. PRSs reflect the additive effects of a large number of common SNPs associated with specific psychiatric and neurodevelopmental disorders [23]. Given that SCZ is clinically and genetically heterogeneous and shares disorder-specific genetic factors with ADHD and ASD, we hypothesized that PRSs differentiating ADHD from ASD would be associated with cognitive impairments and abnormalities in regional surface area and cortical thickness (e.g., decreased cortical thickness and surface area in the frontal cortex) in patients with SCZ. In this study, we investigated whether PRSs based on the GWAS differentiating ADHD from ASD, i.e., neurodevelopmental disorder-specific PRSs, were associated with brain cortical structures as well as cognitive function in patients with SCZ.

## Methods

### **Discovery GWAS differentiating ADHD from ASD (ASD**vs.**ADHD)**

To identify disorder-specific (ASD vs. ADHD) variants, including their *p* values and odds ratios (ORs), we utilized the publicly available GWAS dataset (ASD vs. ADHD) [16] from iPSYCH and the PGC (https://ipsych.dk/en/research/downloads). This dataset comprised 9,315 individuals with ASD and 11,964 individuals with ADHD, all of whom were of European descent [16]. ASD patients in iPSYCH through the Danish Psychiatric Central Research Register (DPCRR) were diagnosed by a psychiatrist according to the ICD, Tenth Revision (ICD-10), and included diagnoses of childhood autism developmental disorders (F84.0), atypical autism (F84.1), Asperger’s syndrome (F84.5), other pervasive developmental disorders (F84.8), and pervasive developmental disorder, unspecified (F84.9). ASD diagnosis of patients in the PGC was achieved through standard research tools, such as the Autism Diagnostic Interview-Revised (ADI-R), the Autism Diagnostic Observation Schedule (ADOS) or international diagnostic criteria [DSM, Fourth Edition (DSM-IV); ICD, Ninth Revision (ICD-9); or ICD-10], and expert clinical consensus. ADHD patients in iPSYCH through the National Psychiatric Central Research Register were diagnosed by psychiatrists at a psychiatric hospital according to the ICD-10 (F90.0). ADHD patients in the PGC were diagnosed according to the DSM, Third Edition Reserved (DSM-III-R), DSM-IV, or ICD-10. Individuals with moderate to severe mental retardation (ICD-10: F71-F79) were excluded. The participants were genotyped using different arrays for each study site. Quality control (QC) and imputation were performed for each dataset separately, as previously described [16]. For PRS analysis, SNPs with low imputation quality (INFO < 0.6) and low genotyping rates of whole subjects (< 60%) were excluded, ultimately resulting in the retention of 6,168,324 SNPs.

### Target sample description

Our target sample included 168 SCZ patients (mean age ± SD: 45.1 ± 13.6 years, 76 males/92 females), all of whom were of Japanese descent with no known first- or second-degree relatives with SCZ. The demographic details are summarized in Table 1. Patients were recruited from the Schizophrenia Non-Affected Relative Project (SNARP) [24–31], and were diagnosed using unstructured clinical interviews, medical records, and clinical consensus according to the criteria in the DSM-5. The exclusion criteria included neurological or medical conditions affecting the central nervous system, such as atypical headache, head trauma, chronic lung disease, kidney or liver diseases, active cancer, cerebrovascular disease, thyroid disorders, epilepsy, seizures, substance-related disorders, steroid use, or intellectual disability. Only two SCZ patients had comorbid ASD, and no SCZ patients had comorbid ADHD. Clinical symptoms were assessed with the Positive and Negative Syndrome Scale (PANSS), and premorbid IQ was measured using the Japanese version of the National Adult Reading Test (JART) [32]. Written informed consent was obtained from all participants after the procedures were thoroughly explained. This study was performed in accordance with the World Medical Association’s Declaration of Helsinki and was approved by the Research Ethics Committees of Gifu University and Kanazawa Medical University.

**Table 1:** Demographic and clinical characteristics of the 168 patients with SCZ

	Mean	SD		Median [Range]
**Age (years)**	45.1	13.6		44.0 [17–83]
**Sex (male/female)**	76/92			-
**Education (years)**	12.5	2.2		12.0 [9-17]
**Premorbid IQ**	98.3	11.0		98.1 [76.9-124.1]
**Age at onset (years)**	27.0	11.0		25.0 [8–68]
**Duration of illness (years)**	17.9	12.4		18.0 [0–56]
**CPZeq (mg/day)**	523.3	511.6		400.0 [0-2859]
**Typical CPZeq (mg/day)**	56.2	204.0		0.0 [0-1723]
**Atypical CPZeq (mg/day)**	467.1	452.7		376.5 [0-2609]
**BPDeq (mg/day)**	0.8	2.3		0.0 [0–23]
**DZPeq (mg/day)**	5.7	8.8		0.0 [0–42]
**PANSS positive symptoms**	16.4	6.2		15.0 [7-36]
**PANSS negative symptoms**	19.3	7.0		19.0 [7-36]

The means and standard deviations (SDs) of patients with schizophrenia are presented. CPZeq, chlorpromazine equivalent; BPDeq, biperiden equivalent; DZPeq, diazepam equivalent; PANSS, Positive and Negative Syndrome Scale. Complete demographic information was not obtained for all participants (estimated premorbid IQ, *n* = 165)

### Genotyping and imputation

A detailed description of the genotyping, QC and imputation procedures applied in larger samples, including the current target sample and previous participants (*n* = 420), to increase the reliability has been provided previously [26, 28]. Briefly, peripheral venous blood was collected from the target subjects, and genomic DNA was extracted from the whole-blood samples. Genotyping was performed using the Infinium OmniExpressExome-8 v1.4 or v1.6 BeadChips (Illumina, San Diego, CA, USA). We excluded SNPs that (i) were duplicated or ambiguous, (ii) were localized on sex chromosomes or mitochondria, (iii) deviated from Hardy-Weinberg equilibrium (HWE) (*p* < 1.0 × 10^− 5^), or (iv) had a low minor allele frequency (MAF) < 0.001. Genotype imputation was performed using the 1000 Genomes Project Phase 3 dataset as a reference panel. To obtain a highly informative SNP set, insertion‒deletion polymorphisms were excluded, and SNPs with high imputation quality (> 0.9) were retained for PRS analysis. Ultimately, 8,538,535 SNPs were retained.

### PRS calculations

To remove SNPs that were in linkage disequilibrium (LD) in the target sample, the SNPs were pruned using a pairwise *r*^*2*^ threshold of 0.25 and a window size of 200 SNPs using PLINK v1.9 [26–30]. After pruning, 1,510,671 independent SNPs remained. Because common disorders are influenced by numerous SNPs across the genome, we then calculated PRSs constructed from alleles showing a nominal association with individuals (ASD vs. ADHD) in the discovery GWAS with the following *P*_*Threshold*_ (*P*_*T*_) cutoff values: *P*_*T*_<1.00 × 10^− 3^, *P*_*T*_<0.01, *P*_*T*_<0.05, *P*_*T*_<0.1, *P*_*T*_<0.2, *P*_*T*_<0.5 and *P*_*T*_≤1. The *P*_*T*_ value represents the *p*-value threshold for including SNPs in the PRS calculation. Since polygenic disorders are likely to involve a large number of SNPs, we employed more relaxed *P*_*T*_ values to capture a broader range of SNPs potentially associated with the disorder. For each individual included in the target sample, a PRS was calculated by weighing the scores for “risk variants” by the logarithm of the OR (logOR) observed in the discovery dataset. The score, consisting of the number of risk variants for differentiating ADHD from ASD (0, 1, or 2) multiplied by the logarithm of the OR, was summed over all of the SNPs in seven *P*_*T*_-SNP sets for each individual in the target sample. A higher PRS indicated a greater risk of ADHD and a lower risk of ASD, while a lower PRS indicated a greater risk of ASD and a lower risk of ADHD.

### Cognitive performance

Cognitive performance in 145 SCZ patients was assessed in four domains—verbal comprehension (VC), perceptual organization (PO), working memory (WM), and processing speed (PS)—using the Japanese version of the Wechsler Adult Intelligence Scale, third edition (WAIS-III) [33]. These cognitive domains were adjusted for age.

### MRI procedure and segmentation of surface area and cortical thickness

Brain magnetic resonance imaging (MRI) was performed on 126 SCZ patients using a Siemens 3T Magnetom Trio, a Tim System (Siemens, Erlangen, Germany). High-resolution T1-weighted images were acquired with a 3D magnetization-prepared rapid gradient echo (MP-RAGE) sequence (TR = 1420 ms, inversion time = 800 ms, echo time = 2.08 ms, flip angle = 9°, resolution = 1 × 1 × 1 mm^3^, matrix size = 256 × 256), yielding 192 contiguous 1 mm thick slices in the sagittal plane [34–40]. We obtained high-resolution T1-weighted images with good contrast between gray matter and white matter in our scanning environment. We screened out subjects with MRI abnormalities, such as infarcts, hemorrhages, or brain tumors, and images with motion or metal artifacts prior to inclusion in this study. Next, the T1-weighted images were processed with FreeSurfer v6.0 using the software package’s default automated reconstruction procedure (‘recon-all’; http://surfer.nmr.mgh.harvard.edu), and surface area and cortical thickness were segmented in 34 brain regions in the right and left hemispheres. These procedures were fully automated and did not involve any manual editing of the images with tissue misclassification. All cortical parcellations were defined from the Desikan–Killiany atlas. Segmented surface area and cortical thickness were visually inspected using a 3D slicer, and there were no subjects with obviously poor segmentation.

### Statistical analysis

All the statistical analyses were performed using IBM SPSS Statistics 28.0 software (IBM Japan, Tokyo, Japan). We explored the association of PRSs differentiating ADHD from ASD at *P*_*T*_<0.5 with cognitive performance in our target SCZ patients using linear regression analyses with cognitive function as the dependent variable, PRSs differentiating ADHD from ASD as the independent variable, and age and sex as covariates, although age was adjusted for when calculating cognitive domain scores. Following a previous study [41], we investigated the effects of PRSs differentiating ADHD from ASD based on each *P*_*T*_ on cortical structures (surface area and cortical thickness) in our target SCZ patients using linear regression analyses with cortical structures as the dependent variable; PRSs differentiating ADHD from ASD based on each *P*_*T*_ as the independent variable; and age, sex, age^2^, age×sex, and intracranial volume (ICV) as covariates for surface area or age and sex as covariates for cortical thickness. The proportion of the variance for each cortical structure explained by the PRSs was indicated by the adjusted *R*^*2*^. To determine the variance explained only by the PRS, we subtracted the adjusted *R*^*2*^ for the covariates alone from those of the models. The nominal two-tailed significance level for all the statistical tests was set at *p* < 0.05. A conservative Bonferroni-corrected *p* value threshold of *p* < 1.47 × 10^− 3^ (= 0.05/34 brain regions) was used in the PRS analysis to avoid type I errors.

## Results

### **Associations of the PRSs differentiating ADHD from ASD (ADHD**vs.**ASD) with cognitive impairments in patients with SCZ**

**Fig. 1:**
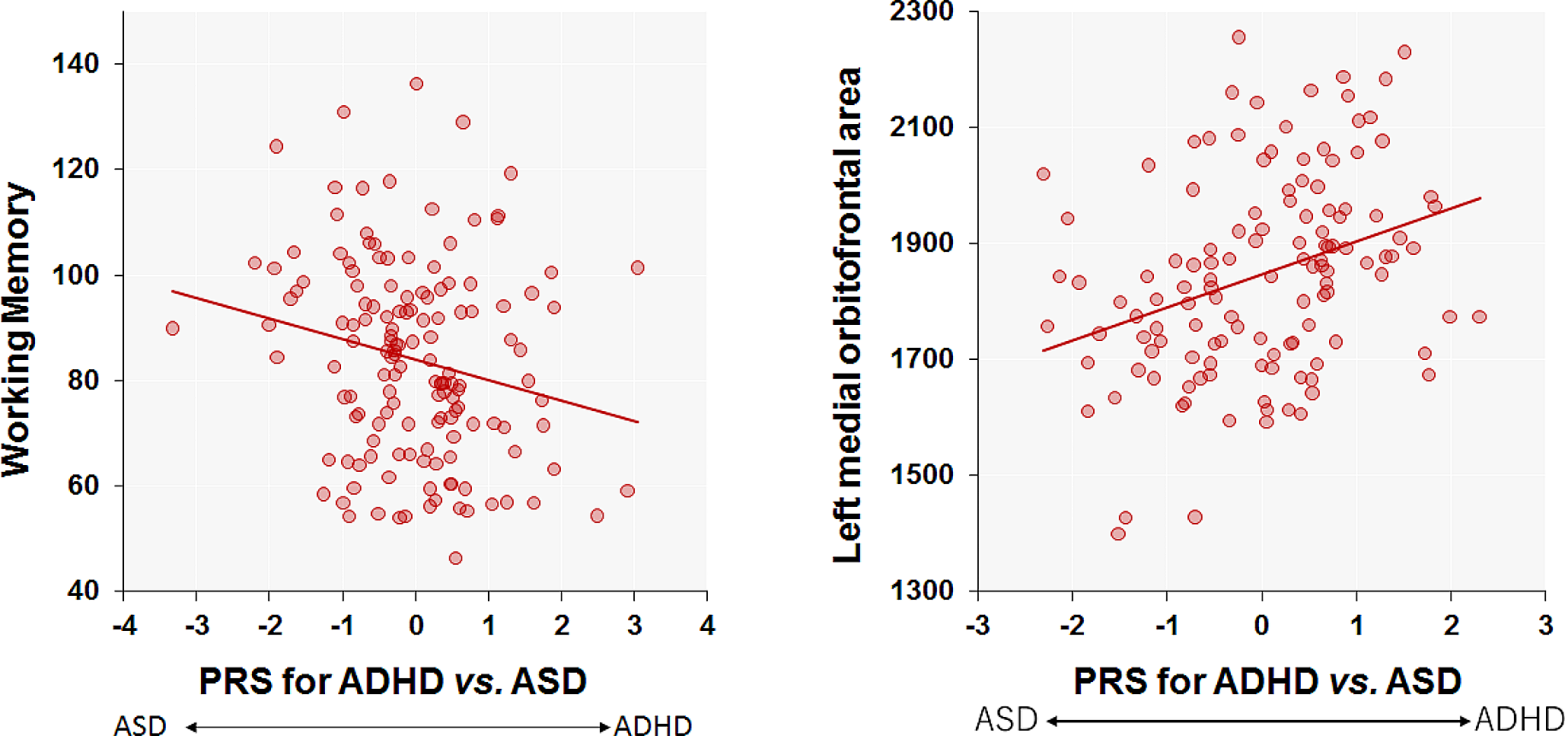
Correlations of the PRSs differentiating ADHD from ASD (ASD vs. ADHD) with working memory and the left medial orbitofrontal area in patients with SCZ. A higher PRS indicated a greater risk of ADHD and a lower risk of ASD, while a lower PRS indicated a greater risk of ASD and a lower risk of ADHD. Working memory scores and the left medial orbitofrontal area were corrected for confounding factors as covariates. The PRS differentiating ADHD from ASD was *z*-transformed. PRS, polygenic risk score; ADHD, attention-deficit hyperactivity disorder; ASD, autism spectrum disorder, SCZ, schizophrenia

Consistent with the negative genetic correlation between the factor differentiating ADHD from ASD and cognitive impairments [16], we observed the association of the PRS differentiating ADHD from ASD at *P*_*T*_<0.5 with four cognitive performances—VC, PO, WM, and PS—in SCZ patients (*p* < 0.05). Notably, a higher PRS (indicating a greater risk of ADHD and a lower risk of ASD) was significantly correlated with impaired WM in SCZ patients (Fig. 1, *beta*=-0.21, *p* = 0.012). However, no significant correlations were found between the PRS and the other three cognitive domains (*p* > 0.05).

### Associations of the PRSs differentiating ADHD from ASD with surface areas in patients with SCZ

**Fig. 2:**
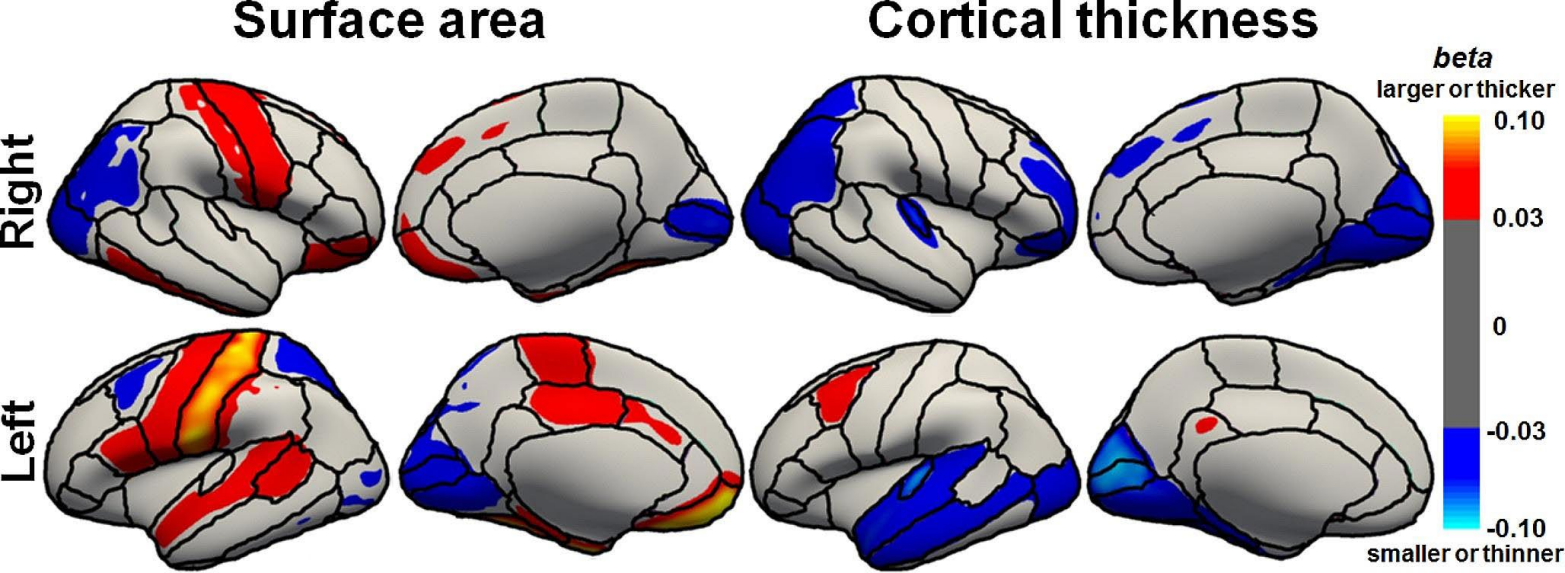
Effects of PRSs differentiating ADHD from ASD at *P*_*T*_<0.5 on surface area and cortical thickness in 34 brain regions in each hemisphere in patients with SCZ. Effect sizes (*beta* values) are represented as a heatmap. Red represents a larger surface area or thicker cortical thickness, and blue represents a smaller surface area or thinner cortical thickness in patients with a greater PRS

**Fig. 3:**
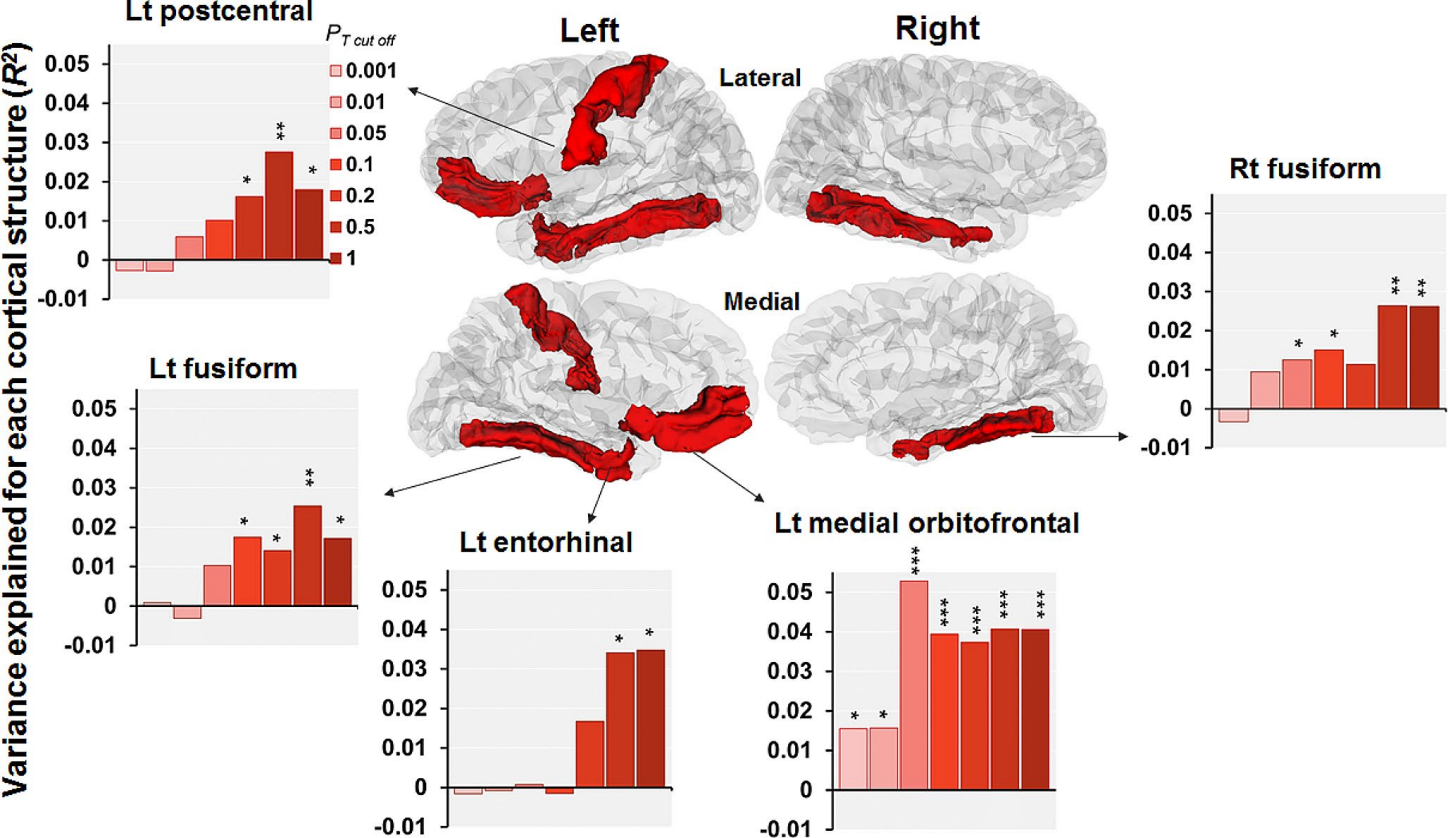
Effects of the PRSs differentiating ADHD from ASD at various thresholds (from *P*_*T cutoff*_<0.001 to *P*_*T cutoff*_<1) on surface areas in patients with SCZ. Five surface areas that showed marginally significant correlations with the PRSs differentiating ADHD from ASD at *P*_*T*_<0.5 (*p* < 0.05) are highlighted. Each y-axis shows the adjusted *R*^*2*^, indicating the explanatory power of the model. Red indicates the association of the lower PRS (indicating a higher risk of ASD) with the decreased surface area. *p < 0.05, ** *p* < 0.01, *** *p* < 1.47 × 10^− 3^

We next investigated the associations of the PRS differentiating ADHD from ASD at *P*_*T*_<0.5 with cortical surface area in SCZ patients (Fig. 2). The PRSs differentiating ADHD from ASD at *P*_*T*_<0.5 showed a nominal positive correlation with five surface areas: the left medial orbitofrontal area, left entorhinal area, left fusiform area, left postcentral area, and right fusiform area (*p* < 0.05, Figs. 2 and 3). Specifically, a lower PRS (indicating a greater risk of ASD and a lower risk of ADHD) was significantly associated with a decreased left medial orbitofrontal area (*beta* = 0.21, *p* = 8.29 × 10^− 4^) and marginally associated with a decreased left entorhinal area (*beta* = 0.21, *p* = 0.025), left postcentral area (*beta* = 0.18, *p* = 7.52 × 10^− 3^), right fusiform area (*beta* = 0.17, *p* = 6.64 × 10^− 3^), and left fusiform area (*beta* = 0.17, *p* = 7.77 × 10^− 3^) (Fig. 2).

Additionally, we examined the effect of PRSs differentiating ADHD from ASD at various *P*_*T*_ levels (from *P*_*T*_<0.001 to *P*_*T*_≤1) on these five surface areas (Fig. 3). The PRSs differentiating ADHD from ASD were significantly positively associated with the left medial orbitofrontal area at several *P*_*T*_ levels (*p* < 1.47 × 10^− 3^, from *P*_*T*_<0.05 to *P*_*T*_≤1, a maximum at *P*_*T*_<0.05: *R*^*2*^ = 0.053, *beta* = 0.24, *p* = 1.48 × 10^− 4^) and nominally positively associated with the left medial orbitofrontal area at *P*_*T*_<0.01 and 0.001 (1.47 × 10^− 3^<*p* < 0.05). A lower PRS differentiating ADHD from ASD at *P*_*T*_ <0.05 was significantly correlated with a smaller left medial orbitofrontal area in patients with SCZ (Fig. 1). Among the other four surface areas, PRSs at several *P*_*T*_ levels were nominally positively associated with the left entorhinal area, left fusiform area, left postcentral area, and right fusiform area (1.47 × 10^− 3^<*p* < 0.05). However, these associations between the PRSs and the four surface areas did not remain significant after Bonferroni correction (*p* > 1.47 × 10^− 3^). The left medial orbitofrontal area was not significantly correlated with WM in our patients (*p* > 0.05), suggesting independent associations of PRSs differentiating ADHD from ASD with WM and this particular cortical area.

### Associations of the PRSs differentiating ADHD from ASD with cortical thickness in patients with SCZ

**Fig. 4:**
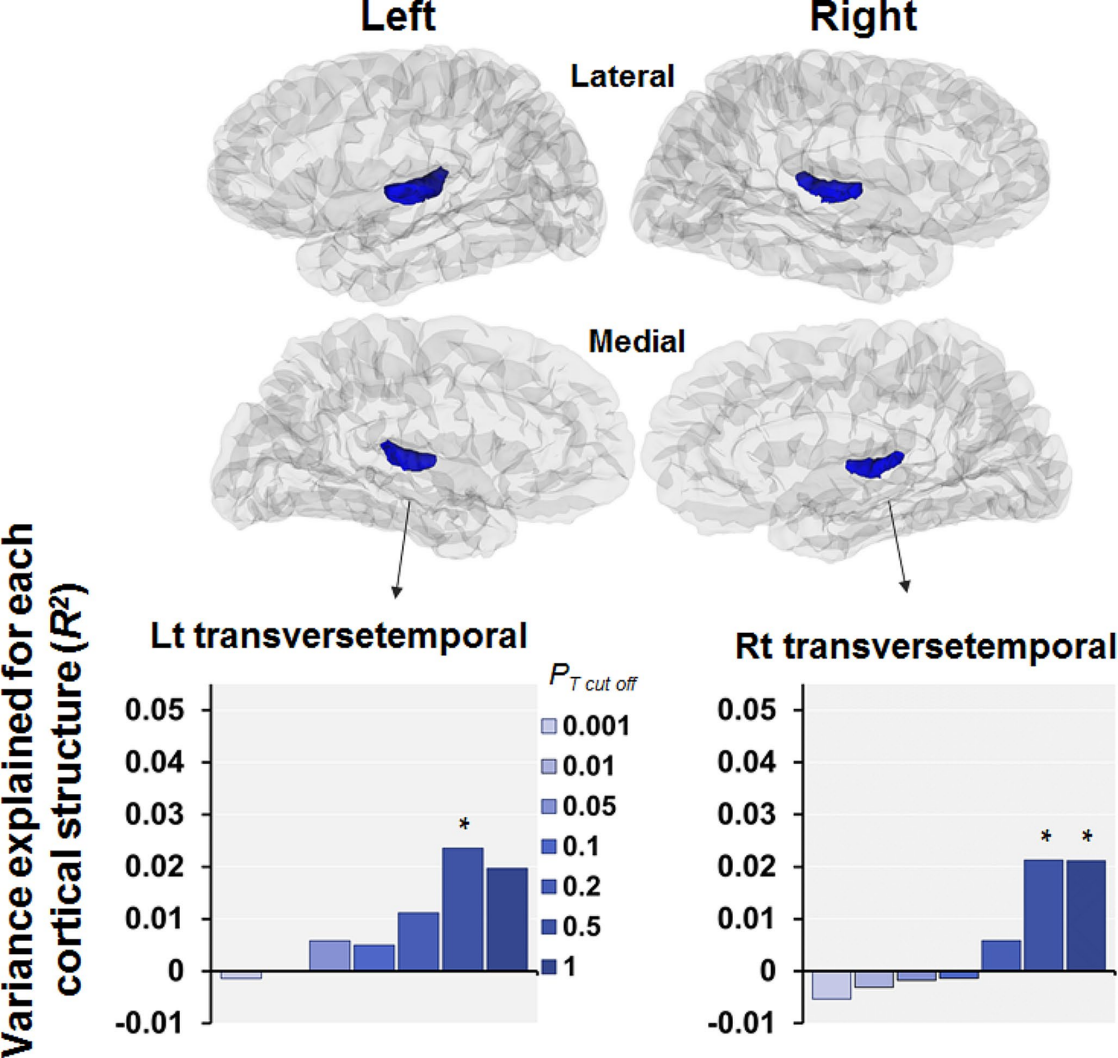
Effects of the PRSs differentiating ADHD from ASD at various thresholds (from *P*_*T cutoff*_<0.001 to *P*_*T cutoff*_≤1) on cortical thickness in patients with SCZ. Two cortical thicknesses showing marginally significant correlations with the PRSs differentiating ADHD from ASD at *P*_*T*_<0.5 (*p* < 0.05) are highlighted. Each y-axis shows the adjusted *R*^*2*^, indicating the model’s explanatory power. Blue highlights the associations of a higher PRS (indicating a greater risk of ADHD) with thinner cortical thickness. * *p* < 0.05

We investigated the associations of PRSs differentiating ADHD from ASD at *P*_*T*_<0.5 with cortical thickness in patients with SCZ (Figs. 2 and 4). We found that PRSs differentiating ADHD from ASD at *P*_*T*_<0.5 were marginally correlated with two cortical thicknesses, the bilateral transverse temporal thickness, in SCZ patients (*p* < 0.05, Figs. 2 and 4). The PRS was marginally negatively associated with the left transverse temporal thickness (*beta*=-0.17, *p* = 0.039) and right transverse temporal thickness (*beta*=-0.17, *p* = 0.045). There were no significant associations between PRS and other cortical thicknesses (*p* > 0.05).

Further analysis of the associations of PRSs differentiating ADHD from ASD at different *P*_*T*_ levels (from *P*_*T*_<0.001 to *P*_*T*_≤1) with bilateral transverse temporal thickness in patients with SCZ (Fig. 4) revealed marginal associations of higher PRSs (indicative of higher ADHD risk) with decreased right transverse temporal thickness at *P*_*T*_<0.5 and *P*_*T*_≤1 (*p* < 0.05, a maximum at *P*_*T*_<0.5: *R*^*2*^ = 0.021, *beta*=-0.17, *p* = 0.045) and left transverse temporal thickness at *P*_*T*_<0.5 only (*R*^*2*^ = 0.024, *beta*=-0.17, *p* = 0.039). However, these associations did not remain significant after correction for multiple testing (*p* > 1.47 × 10^− 3^).

## Discussion

To our knowledge, this is the first study to investigate the association of PRSs differentiating ADHD from ASD with both cortical structures and cognitive performance in patients with SCZ. Consistent with our hypothesis, a higher PRS (indicating a greater risk of ADHD) was associated with impaired cognitive function in SCZ patients. A lower PRS (indicating a greater risk of ASD) was associated with smaller surface areas in five regions (left medial orbitofrontal, left entorhinal, left postcentral, and bilateral fusiform) in SCZ patients. A greater PRS (indicating a greater risk of ADHD) was associated with thinner cortical thicknesses in two regions (bilateral transverse temporal). Notably, the association between the PRS and the left medial orbitofrontal area remained significant even after correcting for multiple testing. These findings suggest that the PRS differentiating ADHD from ASD might reflect cognitive dysfunction and specific cortical structures in SCZ patients, and patients with SCZ who are clinically and genetically heterogeneous might be stratified by the PRS.

Consistent with previous findings on the genetic correlation between ADHD and ASD differentiation and cognitive dysfunctions [16], we identified a genetic correlation between the PRS differentiating ADHD from ASD and cognitive impairment, particularly in WM, in SCZ patients. This finding is consistent with clinical observations that individuals with ADHD typically exhibit lower cognitive function, especially in WM, than do those with ASD [42–44]. SCZ is characterized by severe cognitive impairments, notably WM [45, 46]. These clinical cognitive findings in individuals with ADHD, ASD and SCZ might be supported by the genetic correlations: SCZ and ADHD both show negative genetic correlations with cognitive functions [12, 17], while ASD shows a positive genetic correlation with cognitive functions [11]. Additionally, the genetic factor that differentiates ADHD from ASD is negatively correlated with cognitive functions [16].

The current study demonstrated that a lower PRS differentiating ADHD from ASD (indicating a greater risk of ASD) was significantly correlated with a smaller left medial orbitofrontal area in SCZ patients. Although SCZ patients typically have a smaller medial orbitofrontal surface area than healthy individuals [19], studies on individuals with ASD have shown heterogeneous results [18, 21, 47, 48]. Some studies have shown that individuals with ASD have a smaller medial orbitofrontal surface area than healthy individuals [47, 48], while others have reported no difference between individuals with ASD and healthy individuals [18, 21]. The orbitofrontal cortex is important for decision-making, goal-directed behavior, and emotional processing of faces and is associated with emotional and social behaviors [49–52]. Damage to the orbitofrontal cortex caused by tumors, hemorrhage, ischemic stroke, or aneurysm rupture leads to irrational behavior, as individuals are unable to properly predict the consequences of choosing things in the social environment [51–53]. Individuals with SCZ and ASD often display impaired goal-directed decision-making [54–57] and a decreased ability to recognize emotions in others’ faces and make social judgments [58–62]. Our findings suggest that the PRS for differentiating ASD from ADHD might be related to social dysfunctions via the decreased medial orbitofrontal surface area in SCZ patients.

Although we focused on the associations of PRSs differentiating ADHD from ASD with brain cortical structures, it is important to consider the potential involvement of PRSs in determining shared liability of ADHD and ASD in cortical structures, particularly the orbitofrontal area, in SCZ patients. While we further explored these relationships, no significant correlations between the PRS for shared liability to ADHD and ASD and any cortical structures, including cortical thickness and surface area, were found (*p* > 1.47 × 10^− 3^).

Although it is not the main purpose of our study, to provide a baseline for comparison, we additionally explored the influences of PRSs differentiating ASD from ADHD on cognitive functions and alterations in cortical structures in 195 psychiatrically and neurodevelopmentally healthy individuals (the subject recruitment was described in previous studies [24–31]). A higher PRS, indicating a greater risk of ADHD, at *P*_*T*_<0.5, was nominally associated with decreased surface area of the transverse temporal region (*beta*=-0.16, *p* = 0.020) and decreased cortical thickness in the left postcentral gyrus (*beta*=-0.14, *p* = 0.049) in 175 healthy individuals, while these marginal associations did not remain significant after correction for multiple testing (*p* > 1.47 × 10^− 3^). In contrast, there were no significant associations between the PRSs and four cognitive performances—VC, PO, WM, and PS— in 153 healthy individuals (*p* > 0.05). The fact that we assessed healthy subjects who were carefully screened to exclude psychiatric and neurodevelopmental patients [24–31] might have contributed to the lack of these associations in the healthy subjects. Similar associations to those in SCZ patients might be obtained in the general population, including patients with psychiatric or neurodevelopmental disorders.

There are limitations in interpreting our findings. This study focused exclusively on SCZ patients, limiting the scope of understanding whether these genetic factors also contribute to stratification in other psychiatric disorders. Although we found associations between higher and lower PRSs differentiating ADHD from ASD with impaired working memory and reduced left medial orbitofrontal area, respectively, the relationships of these PRSs with the risk and progression of SCZ remain unclear. Future studies should include other psychiatric disorder groups to better elucidate the impact of these PRSs on individuals with SCZ. This would enhance our understanding of the distinct and overlapping genetic contributions to cognitive and cortical abnormalities in SCZ compared to other psychiatric disorders.

In conclusion, our study revealed associations between genetic factors differentiating ADHD from ASD and both cortical structures and cognitive performance in SCZ patients. These findings suggest that the clinical and genetic heterogeneity in SCZ patients might be influenced by genetic factors associated with other neurodevelopmental and psychiatric disorders beyond SCZ.

## Data Availability

No datasets were generated or analysed during the current study.
